# 6,8-Dihydr­oxy-3-methyl­isocoumarin

**DOI:** 10.1107/S1600536809008083

**Published:** 2009-03-11

**Authors:** Chang-lun Shao, Lei Han, Chun-yuan Li, Zhen Liu, Chang-yun Wang

**Affiliations:** a School of Medicine and Pharmacy, Ocean University of China, Qingdao, Shandong 266003, People’s Republic of China; b College of Science, South China Agricultural University, Guangzhou, Guangdong 510642, People’s Republic of China; c College of Chemistry and Chemical Engineering, Luoyang Normal University, Luoyang, Henan 471022, People’s Republic of China

## Abstract

The title compound, C_10_H_8_O_4_, was isolated from the fermentation culture of the endophytic fungus *Cephalo­sporium* sp. In the crystal structure, mol­ecules are connected into a one-dimensional chain along [101] by inter­molecular O—H⋯O hydrogen bonds involving the hydroxyl and carbonyl functionalities. The chains are linked by non-classical C—H⋯O inter­actions, forming extended two-dimensional layers approximately parallel to (11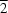).

## Related literature

For new bioactive secondary metabolites from marine fungi, see: Shao *et al.* (2007 ▶). For the investigation of an endophytic strain *Cephalosporium *sp., see: Wei *et al.* (2008 ▶); Hemingway *et al.* (1977 ▶); Kendall *et al.* (1989 ▶). For crystal structures with non-classical C—H⋯O inter­actions, see: Nangia (2002 ▶).
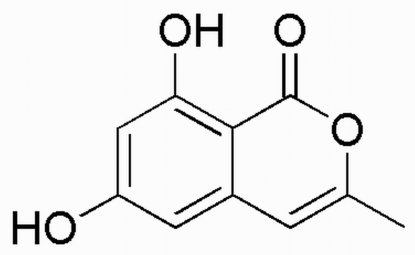


## Experimental

### 

#### Crystal data

C_10_H_8_O_4_
*M* *_r_* = 192.16
Monoclinic, 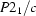

*a* = 3.8201 (7) Å
*b* = 15.710 (3) Å
*c* = 14.196 (2) Å
β = 92.668 (2)°
*V* = 851.1 (3) Å^3^
*Z* = 4
Mo *K*α radiation
μ = 0.12 mm^−1^
*T* = 291 K
0.27 × 0.20 × 0.19 mm

#### Data collection

Bruker APEXII CCD diffractometer
Absorption correction: multi-scan (*SADABS*; Sheldrick, 1996 ▶) *T*_min_ = 0.969, *T*_max_ = 0.978
4781 measured reflections
1586 independent reflections
1272 reflections with *I* > 2σ(*I*)
*R*_int_ = 0.021

#### Refinement

*R*[*F* ^2^ > 2σ(*F* ^2^)] = 0.034
*wR*(*F* ^2^) = 0.094
*S* = 1.04
1586 reflections
131 parameters
H-atom parameters constrained
Δρ_max_ = 0.18 e Å^−3^
Δρ_min_ = −0.14 e Å^−3^

### 

Data collection: *APEX2* (Bruker, 2004 ▶); cell refinement: *SAINT* (Bruker, 2004 ▶); data reduction: *SAINT*; program(s) used to solve structure: *SHELXS97* (Sheldrick, 2008 ▶); program(s) used to refine structure: *SHELXL97* (Sheldrick 2008 ▶); molecular graphics: *SHELXTL* (Sheldrick 2008 ▶); software used to prepare material for publication: *SHELXTL* and *PLATON* (Spek, 2009 ▶).

## Supplementary Material

Crystal structure: contains datablocks global, I. DOI: 10.1107/S1600536809008083/bh2219sup1.cif

Structure factors: contains datablocks I. DOI: 10.1107/S1600536809008083/bh2219Isup2.hkl

Additional supplementary materials:  crystallographic information; 3D view; checkCIF report

## Figures and Tables

**Table 1:** Hydrogen-bond geometry (Å, °)

*D*—H⋯*A*	*D*—H	H⋯*A*	*D*⋯*A*	*D*—H⋯*A*
O4—H4⋯O2^i^	0.82	1.96	2.7225 (15)	155
C5—H5⋯O4^ii^	0.93	2.60	3.4659 (19)	155

Symmetry codes: (i) 
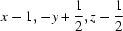
; (ii) 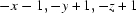.

**Fig. 1.**
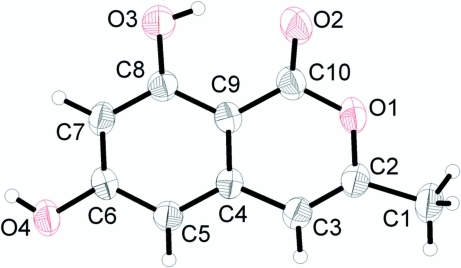
View of the title molecule with atom numbering scheme and 30% probability displacement ellipsoids for non-hydrogen atoms.

**Fig. 2.**
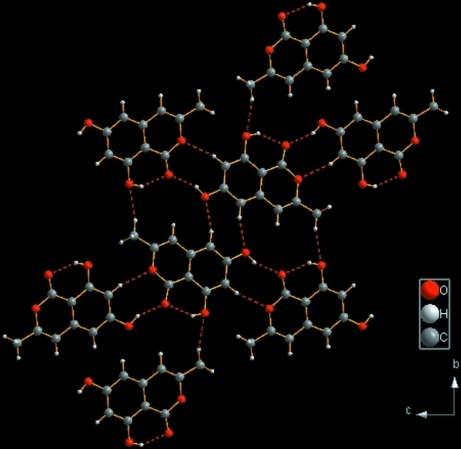
View of the 2D layers formed by intermolecular O—H···O hydrogen bonds and weak non-conventional intermolecular C—H···O interactions.

## supplementary crystallographic information

### Comment

Endophytic fungi have proven to be a rich source of novel structural compounds with interesting biological activities and a high level of biodiversity. In the course of our search for new or bioactive secondary metabolites from the marine fungi (Shao *et al.*, 2007). we have investigated an endophytic strain *Cephalosporium sp*. (Wei *et al.*, 2008). The title compound was previously isolated from the organic extracts of the fungus *Ceratocystis minor* (Hemingway *et al.*, 1977), and elucidated on the basic of spectroscopic analysis (Kendall *et al.*, 1989). Herein, the title compound was isolated from the fermentation culture of the endophytic fungus *Cephalosporium sp*., and its crystal structure is reported.

The asymmetric unit of the title compound contains one independent molecule (Fig. 1), in which the bond lengths and angles are within the expected ranges. The structural analysis reveals that the most relevant feature is the arrangement of the molecules, which are connected to form a one-dimensional chain along the [101] direction, by the formation of intermolecular O—H···O hydrogen bonds. Furthermore, weak non-conventional intermolecular C—H···O interactions are observed (Nangia, 2002), in which C5—H5 is a donor and O4 is an acceptor. These interactions consolidate the crystal packing. Details of hydrogen bonds are given in Table 1.

### Experimental

A strain of fungus *Cephalosporium sp*. (No. 2090) was deposited in the School of Chemistry and Chemical Engineering, Sun Yat-sen University, Guangzhou, People's Republic of China. Culture conditions: GYT medium (glucose 10 g/L, peptone 2 g/L, yeast extract 1 g/L, NaCl 2.5 g/L) and incubation at 298 K for 4 weeks. The cultures (70 L) were filtered through cheesecloth. The filtrate was concentrated to 3 L below 323 K, extracted five times by shaking with an equal volume of ethyl acetate. The extract was evaporated under reduced pressure below 323 K. The combined organic extracts were chromatographed on silica-gel, eluting with petroleum ether/ethyl acetate, to yield the title compound. Crystals were obtained by evaporation of an ethyl acetate solution.

### Refinement

All H atoms were positioned geometrically and treated as riding, with C—H bond lengths constrained to 0.93 (aromatic CH), 0.96 (methyl CH_3_), and 0.82 Å (hydroxyl OH), and with *U*_iso_(H) = 1.5*U*eq(carrier atom) or for CH_3_ and OH groups and *U*_iso_(H) = 1.2*U*eq(carrier C) otherwise.

### Crystal data

**Table:**

C_10_H_8_O_4_	*F*(000) = 400
*M**_r_* = 192.16	*D*_x_ = 1.500 Mg m^−^^3^
Monoclinic, *P*2_1_/*c*	Mo *K*α radiation, λ = 0.71073 Å
Hall symbol: -P 2ybc	Cell parameters from 1738 reflections
*a* = 3.8201 (7) Å	θ = 2.6–25.5°
*b* = 15.710 (3) Å	µ = 0.12 mm^−^^1^
*c* = 14.196 (2) Å	*T* = 291 K
β = 92.668 (2)°	Block, yellow
*V* = 851.1 (3) Å^3^	0.27 × 0.20 × 0.19 mm
*Z* = 4	

### Data collection

**Table:**

Bruker APEXII CCD diffractometer	1586 independent reflections
Radiation source: fine-focus sealed tube	1272 reflections with *I* > 2σ(*I*)
graphite	*R*_int_ = 0.021
φ and ω scans	θ_max_ = 25.5°, θ_min_ = 2.6°
Absorption correction: multi-scan (*SADABS*; Sheldrick, 1996)	*h* = −4→4
*T*_min_ = 0.969, *T*_max_ = 0.978	*k* = −19→13
4781 measured reflections	*l* = −17→17

### Refinement

**Table:**

Refinement on *F*^2^	Secondary atom site location: difference Fourier map
Least-squares matrix: full	Hydrogen site location: inferred from neighbouring sites
*R*[*F*^2^ > 2σ(*F*^2^)] = 0.034	H-atom parameters constrained
*wR*(*F*^2^) = 0.094	*w* = 1/[σ^2^(*F*_o_^2^) + (0.0441*P*)^2^ + 0.1957*P*] where *P* = (*F*_o_^2^ + 2*F*_c_^2^)/3
*S* = 1.04	(Δ/σ)_max_ < 0.001
1586 reflections	Δρ_max_ = 0.18 e Å^−^^3^
131 parameters	Δρ_min_ = −0.14 e Å^−^^3^
0 restraints	Extinction correction: *SHELXL97* (Sheldrick 2008), Fc^*^=kFc[1+0.001xFc^2^λ^3^/sin(2θ)]^-1/4^
0 constraints	Extinction coefficient: 0.015 (3)
Primary atom site location: structure-invariant direct methods	

### Fractional atomic coordinates and isotropic or equivalent isotropic displacement parameters (Å^2^)

**Table:**

	*x*	*y*	*z*	*U*_iso_*/*U*_eq_	
O1	0.3482 (3)	0.32453 (7)	0.81217 (7)	0.0451 (3)	
O2	0.3544 (3)	0.19366 (7)	0.75748 (8)	0.0572 (4)	
O3	0.0718 (3)	0.15766 (7)	0.58873 (8)	0.0552 (4)	
H3	0.1709	0.1476	0.6400	0.083*	
O4	−0.4021 (3)	0.39231 (7)	0.41364 (7)	0.0487 (3)	
H4	−0.4514	0.3541	0.3759	0.073*	
C1	0.3853 (4)	0.45742 (12)	0.89102 (11)	0.0516 (4)	
H1A	0.3148	0.5160	0.8859	0.077*	
H1B	0.6359	0.4542	0.8987	0.077*	
H1C	0.2819	0.4321	0.9446	0.077*	
C2	0.2659 (4)	0.41080 (10)	0.80372 (10)	0.0395 (4)	
C3	0.1028 (4)	0.44079 (10)	0.72629 (10)	0.0379 (4)	
H3A	0.0505	0.4985	0.7224	0.046*	
C4	0.0042 (3)	0.38590 (9)	0.64774 (9)	0.0318 (3)	
C5	−0.1641 (4)	0.41528 (9)	0.56587 (10)	0.0355 (3)	
H5	−0.2230	0.4725	0.5598	0.043*	
C6	−0.2453 (4)	0.35866 (10)	0.49244 (10)	0.0354 (3)	
C7	−0.1659 (4)	0.27235 (9)	0.50066 (10)	0.0378 (4)	
H7	−0.2235	0.2354	0.4512	0.045*	
C8	−0.0016 (4)	0.24192 (9)	0.58233 (10)	0.0368 (3)	
C9	0.0880 (4)	0.29837 (9)	0.65755 (9)	0.0337 (3)	
C10	0.2651 (4)	0.26818 (10)	0.74166 (10)	0.0401 (4)	

### Atomic displacement parameters (Å^2^)

**Table:**

	*U*^11^	*U*^22^	*U*^33^	*U*^12^	*U*^13^	*U*^23^
O1	0.0531 (7)	0.0503 (7)	0.0308 (5)	0.0018 (5)	−0.0102 (5)	−0.0008 (5)
O2	0.0802 (9)	0.0448 (7)	0.0447 (7)	0.0086 (6)	−0.0182 (6)	0.0060 (5)
O3	0.0804 (9)	0.0330 (6)	0.0503 (7)	0.0065 (5)	−0.0162 (6)	−0.0011 (5)
O4	0.0677 (8)	0.0425 (6)	0.0342 (6)	0.0033 (5)	−0.0174 (5)	−0.0004 (5)
C1	0.0488 (10)	0.0661 (11)	0.0390 (9)	−0.0024 (8)	−0.0060 (7)	−0.0131 (8)
C2	0.0382 (8)	0.0459 (9)	0.0344 (8)	−0.0026 (7)	0.0008 (6)	−0.0058 (6)
C3	0.0414 (8)	0.0372 (8)	0.0350 (8)	−0.0021 (6)	0.0002 (6)	−0.0034 (6)
C4	0.0311 (7)	0.0354 (8)	0.0291 (7)	−0.0033 (6)	0.0016 (6)	−0.0006 (6)
C5	0.0405 (8)	0.0314 (8)	0.0344 (7)	0.0001 (6)	−0.0011 (6)	0.0009 (6)
C6	0.0354 (8)	0.0407 (8)	0.0297 (7)	−0.0013 (6)	−0.0037 (6)	0.0029 (6)
C7	0.0437 (8)	0.0379 (9)	0.0312 (7)	−0.0045 (6)	−0.0047 (6)	−0.0053 (6)
C8	0.0412 (8)	0.0318 (8)	0.0370 (8)	−0.0010 (6)	−0.0011 (6)	−0.0003 (6)
C9	0.0344 (8)	0.0364 (8)	0.0301 (7)	−0.0011 (6)	−0.0005 (6)	0.0019 (6)
C10	0.0436 (9)	0.0422 (9)	0.0339 (8)	0.0007 (7)	−0.0030 (6)	0.0033 (6)

### Geometric parameters (Å, °)

**Table:**

O1—C10	1.3626 (18)	C3—C4	1.4457 (19)
O1—C2	1.3951 (19)	C3—H3A	0.9300
O2—C10	1.2370 (18)	C4—C5	1.3813 (19)
O3—C8	1.3553 (18)	C4—C9	1.417 (2)
O3—H3	0.8200	C5—C6	1.394 (2)
O4—C6	1.3518 (17)	C5—H5	0.9300
O4—H4	0.8200	C6—C7	1.393 (2)
C1—C2	1.493 (2)	C7—C8	1.378 (2)
C1—H1A	0.9600	C7—H7	0.9300
C1—H1B	0.9600	C8—C9	1.418 (2)
C1—H1C	0.9600	C9—C10	1.426 (2)
C2—C3	1.325 (2)		
			
C10—O1—C2	121.61 (11)	C4—C5—C6	119.64 (13)
C8—O3—H3	109.5	C4—C5—H5	120.2
C6—O4—H4	109.5	C6—C5—H5	120.2
C2—C1—H1A	109.5	O4—C6—C7	122.43 (13)
C2—C1—H1B	109.5	O4—C6—C5	116.35 (13)
H1A—C1—H1B	109.5	C7—C6—C5	121.22 (13)
C2—C1—H1C	109.5	C8—C7—C6	119.81 (13)
H1A—C1—H1C	109.5	C8—C7—H7	120.1
H1B—C1—H1C	109.5	C6—C7—H7	120.1
C3—C2—O1	120.81 (13)	O3—C8—C7	118.69 (13)
C3—C2—C1	128.92 (15)	O3—C8—C9	121.23 (13)
O1—C2—C1	110.27 (13)	C7—C8—C9	120.08 (13)
C2—C3—C4	121.57 (14)	C4—C9—C8	119.18 (12)
C2—C3—H3A	119.2	C4—C9—C10	120.08 (13)
C4—C3—H3A	119.2	C8—C9—C10	120.74 (13)
C5—C4—C9	120.07 (12)	O2—C10—O1	115.39 (13)
C5—C4—C3	122.96 (13)	O2—C10—C9	125.66 (14)
C9—C4—C3	116.96 (12)	O1—C10—C9	118.96 (13)
			
C10—O1—C2—C3	0.1 (2)	C5—C4—C9—C8	0.3 (2)
C10—O1—C2—C1	179.78 (13)	C3—C4—C9—C8	−179.69 (13)
O1—C2—C3—C4	−0.1 (2)	C5—C4—C9—C10	179.32 (13)
C1—C2—C3—C4	−179.74 (14)	C3—C4—C9—C10	−0.66 (19)
C2—C3—C4—C5	−179.60 (14)	O3—C8—C9—C4	−179.69 (13)
C2—C3—C4—C9	0.4 (2)	C7—C8—C9—C4	0.5 (2)
C9—C4—C5—C6	−1.1 (2)	O3—C8—C9—C10	1.3 (2)
C3—C4—C5—C6	178.85 (13)	C7—C8—C9—C10	−178.57 (13)
C4—C5—C6—O4	−178.42 (12)	C2—O1—C10—O2	179.49 (13)
C4—C5—C6—C7	1.3 (2)	C2—O1—C10—C9	−0.4 (2)
O4—C6—C7—C8	179.14 (14)	C4—C9—C10—O2	−179.17 (15)
C5—C6—C7—C8	−0.5 (2)	C8—C9—C10—O2	−0.2 (2)
C6—C7—C8—O3	179.79 (14)	C4—C9—C10—O1	0.7 (2)
C6—C7—C8—C9	−0.3 (2)	C8—C9—C10—O1	179.67 (13)

### Hydrogen-bond geometry (Å, °)

**Table:**

*D*—H···*A*	*D*—H	H···*A*	*D*···*A*	*D*—H···*A*
O3—H3···O2	0.82	1.92	2.6426 (16)	146
O4—H4···O2^i^	0.82	1.96	2.7225 (15)	155
C5—H5···O4^ii^	0.93	2.60	3.4659 (19)	155

Symmetry codes: (i) *x*−1, −*y*+1/2, *z*−1/2; (ii) −*x*−1, −*y*+1, −*z*+1.
